# Displacements

**Published:** 1904-05

**Authors:** S. T. Barnett

**Affiliations:** Atlanta, Ga.


### DISPLACEMENTS.

BY S. T. BARNETT, M.D.,
ATLANTA, GA.

The subject is one entirely too broad in its scope to allow of anything more than a mere touching on some of the salient features of these conditions in the time at my disposal. I prefer not to take it up in a stereotyped manner and will, therefore, with some preliminary remarks on the anatomy of the parts, limit myself to the non-operative treatment.

It has for a number of years been my belief that the anatomy of the normal female pelvis has not been understood as thoroughly as it should be. In many ways we have a profuse literature on the parts. The pelvic floor has been worked out more or less thoroughly by a number of investigators; and we have been taught to believe by most lecturers and writers on the subject that this pelvic floor, as described by the majority of men, together with the ligaments, broad, round utero-sacral and vesico-uterine, are the essential elements in the support of the uterus and its appendages. But in describing the pelvic floor, they lay most stress on the levator ani muscle and the fasci as immediately contiguous or overlying this muscle, seemingly ignoring the fact that the uterus is situated on a higher plane than even its attachments, while the center of the muscle swings lower still. They answer this by saying the ligaments are the main support, and yet in describing the support given by the ligaments, they ascribe the main part of it to the round and broad ligaments, laying but minor stress on the utero-sacral, anterio posterior or ovarian. It is a well-known fact that you can cut through almost the entire broad and completely through the round of both sides and still the uterus will not sink deeper into the pelvic cavity. The organ will, it is true, tilt to one or the other side more readily, but the *level of the isthmus*

*remains the same.* The action of these ligaments are then merely as stays. I have carefully read both Gray and Gerrish for a thorough description of these parts. Gray gives an indistinct description, which is not at all satisfying as to what are the real supports of the organ. Gerrish gives even less. That most excellently illustrated work on Operative Gynecology by Kelley goes into the minutest detail, pictorially, in describing the most of the anatomical conditions of the female pelvis, but on the supports of the uterus it is almost silent. I do not believe with Pryor that the support of the uterus is that "The intra-abdominal pressure is uniform under ordinary circumstances." Most of the gynecologies have little but what you find in the anatomies, though both Pazzi and Dudley hint at the utero-sacral ligaments being the most important element. Pazzi, in fact, says it is slung as it were in a sling, with the small end down, and stayed in position by the other ligaments. Williams, in his obstetrics, describes the ligamentum transversale colli of Mackenrodt lying beneath the broad ligament. These, with the utero-sacral ligaments are, to my mind, the essential supports of the uterus. In this connection it must not be lost sight of that normally the uterus lies ante-verted and slightly ante-flexed on the posterior surface of the empty bladder, and, in some measure, derives its normal support from the supports of that organ. The point I want to bring out especially, though, is that this subject of the uterine supports is universally badly exploited in most text-books, and offers a fine field for original anatomical work. And, secondly, if we have not a thorough understanding of the normal uterine supports and their intrinsic value, how can we expect to handle to the best advantage displacements of that organ?

The treatment of these conditions alone would be far more than sufficient to occupy our time. As I said in the beginning, I will speak mainly of non-operative treatment. I fully realize, however, the great and tremendous good that properly directed operative work has done along these lines. The greater part of my medical life has been lived in an atmosphere of surgical relief for these conditions. In consequence of that I have believed that a pessary, as a rule, was a thing to be relegated to the past and not in accordance with up-to-date methods. The cause of this mental condition is readily appreciated. But has not the pendulum swung too far? Are there not many conditions where a properly fitting pessary snugly placed, preceded by a restoration by manipulation to a normal position, the indicated procedure rather than the knife? I have come to believe so, though I wish to enter a plea for a closer scrutiny of our cases, and to see, if possible, if proper manipulations may not accomplish our ends. If we do we will save many a poor woman from the hands of the quacks, who has not the courage to face the operating table, or, having faced it, finds herself no better. I would divide my treatment into the prophylactic and manipulative.

Prophylactic is trying to put the girl or woman in the condition that nature intended her—trying to give her healthy excretory and secretory organs performing their function, eliminating those toxic principles so enfeebling to retain, carrying off the waste products of cell repair, and bringing to all the organs rich, oxygenated, tissue-building blood. There is nothing so conducive to these displacements as faulty nutrition.

I think we physicians lay entirely too little consideration to the dress. The constricting bands of many folds of cloth, etc., at the waist increasing abdominal pressure, and pelvic hyperæmia and decreasing abdmoninal muscle-tone and consequently the gentle intestinal massage so necessary for health, is a large factor in the predisposing causes of these troubles.

When the woman comes to confinement, the scrupulous care during that period, the proper repair of tears, and the after-care of the puerperal period, will often be the determining factor in normal or mal-position. The careful attention to the physiological periods, when the supports are normally weakened, are also most important, and should receive due consideration.

The largest class of gynecological cases necessarily belongs to the surgical. It would be folly to advise manipulative methods as long as the pelvic floor was torn, dragging down, as it does, adjacent structures. It would be almost criminal to try manipulation in associated pus conditions. But in those cases, not due to trauma but to a pathological weakening of the supports from general causes, manipulative methods may be attempted. Yet, even here, we must throw out certain cautions. The age of the patient and her nervous temperament have to be considered, with the length of time the condition has existed. If of too long standing, operative methods will give better results; as also if the nervous condition, due to temperament or age, will preclude her undergoing the necessary manipulations.

By manipulations I mean by manual or instrumental means to replace the uterus in its normal position and retain it there by means of some mechanical support or pessary. I fully agree with Davenport in his excellent paper, "That the pessary used in suitable cases is as logical a method of treatment as a plaster bandage in a sprain or a crutch in lameness."

Of the manipulative methods, I would unhesitatingly condemn that of the sound. This especially as usually practiced, of using an unsterilized instrument, in unsterilized hands, through an unsterilized vagina, often in a soft, easily punctured uterus. Some have used it without accident, but it is a *most dangerous procedure*. The use of Sims' repositor is seldom practiced now. Personally I have seen it used but a few times. It is open to some of the objections of the sound, from likelihood of trauma. Re-position by the knee chest position can sometimes be accomplished with less pain to the patient than any other method, but patients usually object thoroughly to the position, and I have found great difficulty at times in putting them in it. It is more applicable to retroversion. The speculum being introduced, the vagina ballooned, a sponge holder, with sponge, lifts the uterus up and then pushes the cervix back. The bi-manual method has been described so often recently that I will not weary you with the repetition. It is the preferable method.

The uterus having been replaced it is held in place by a suitably selected pessary. The *pessary can no more replace a displacement than the splint can reduce a fracture.* But having replaced it, a suitably fitting one retains it, as a suitable splint retains the fractured bone.

But, having replaced the uterus, fitted the pessary in position, do not do like a doctor I heard of once, who, having placed a pessary in the vagina, told her to report in a month or two. She might have, and probably did have, a cystitis set up, or ulceration or so great discomfort that, rather than retain it, she removed it herself. The pessary then should do its work, should be comfortable and non-irritating.

The objection that has generally been made against the pessary is that, acting as an artificial support, the tissues stretch, gradually requiring larger pessaries to do the work and weakening the natural supports of the uterus. A uterus out of position, however, stretches and weakens the ligaments infinitely more, besides adding to the hyperæmic condition. The pessary holds the uterus in place until the hyperæmia subsides, the ligaments regain their tone and, eventually, the pessary, as the splint, can be discarded. But *do not use a pessary in inflammatory conditions* or when the *uterus is firmly fixed.*

The idea of twenty and forty years ago that all displacement conditions required the pessary, naturally brought the practice into deserved disfavor. The practitioner of that time, whenever in doubt, as he was often, played his pessary as the once time-honored dictum in whist, " Whenever in doubt, play trumps," and frequently with the result that the woman suffered excruciating torture and readily submitted to the terror of the table.

In this I have merely touched on the subject, nor attempted to enter deeply. I would mention massage and electricity, both of which have their advocates and which often may be a great adjuvans in handling these cases, but I have had no personal experience in either.

